# PGRMC1 Elevation in Multiple Cancers and Essential Role in Stem Cell Survival

**DOI:** 10.4236/alc.2015.43006

**Published:** 2016-01-28

**Authors:** Kaia K. Hampton, Rachel Stewart, Dana Napier, Pier Paolo Claudio, Rolf J. Craven

**Affiliations:** 1 Department of Pharmacology and Nutritional Sciences, University of Kentucky, Lexington, KY, USA; 2 Department of Pathology and Laboratory Medicine, University of Kentucky, Lexington, KY, USA; 3 Markey Cancer Center, University of Kentucky, Lexington, KY, USA; 4 Department of Biomolecular Sciences and National Center for Natural Products Research, School of Pharmacy, University of Mississippi, Oxford, MS, USA; 5 Department of Radiation Oncology, University of Mississippi Medical Center, Jackson, MS, USA

**Keywords:** Lung Cancer, Oral Cancer, Head and Neck Cancer, Stem Cells, PGRMC1

## Abstract

Cancer is one of the leading causes of death in America, and there is an urgent need for new therapeutic approaches. The progesterone receptor membrane component 1 (PGRMC1) is a cytoch-rome *b_5_* related protein that binds heme and is associated with signaling, apoptotic suppression and autophagy. PGRMC1 is essential for tumor formation, invasion and metastasis, and is upregulated in breast, colon, lung and thyroid tumors. In the present study, we have analyzed PGRMC1 levels in over 600 tumor sections, including a larger cohort of lung tumors than in previous studies, and report the first clinical analysis of PGRMC1 levels in human oral cavity and ovarian tumors compared to corresponding nonmalignant tissues. PGRMC1 was highly expressed in lung and ovarian cancers and correlated with patient survival. PGRMC1 has been previously associated with drug resistance, a characteristic of cancer stem cells. The stem cell theory proposes that a subset of cancerous stem cells contribute to drug resistance and tumor maintenance, and PGRMC1 was detected in lung-tumor derived stem cells. Drug treatment with a PGRMC1 inhibitor, AG-205, triggered stem cell death whereas treatment with erlotinib and the ERK inhibitor, PD98059, did not, suggesting a specific role for PGRMC1 in cancer stem cell viability. Together, our data demonstrate PGRMC1 as a potential tumor biomarker across a variety of tumors, as well as a therapeutic target for cancer stem cells.

## 1. Introduction

PGRMC1 is induced in a number of cancer types [1], including breast, ovarian and lung cancers, and a small study indicated that PGRMC1 is associated with poor survival in lung adenocarcinoma [2]. PGRMC1 is also expressed in sebaceous carcinomas [3]. PGRMC1 plays a causative role in cancer progression, because *in vitro*, PGRMC1 increases tumor cell proliferation, chemotherapy resistance and invasion, and *in vivo*, PGRMC1 increases tumor growth, angiogenesis and metastasis [4]-[9]. There are a number of potential mechanisms through which PGRMC1 might promote tumor growth. PGRMC1 associates with the epidermal growth factor receptor (EGFR) and regulates susceptibility to the EGFR inhibitor erlotinib by increasing plasma membrane pools of EGFR [10]. PGRMC1 also increases EGFR levels in Zebrafish [11]. In lung cancer cells, the EGFR-PGRMC1 complex drives invasion, at least in part, by activating matrix metalloproteinases [12].

PGRMC1 is also detected in the nucleus in some cell types, where it regulates transcription [13] and in the centromeric region of chromosomes during oocyte meiosis [14] [15]. PGRMC1 also localizes to the actin cytoskeleton and binds actin. In lung cancer cells, the prominent localization for PGRMC1 is cytoplasmic puncta, including early endosomes, and numerous groups have reported similar findings in other cell types. Finally, PGRMC1 is secreted by lung cancer cells, where it has a pro-proliferative function, and is detected in the plasma of lung cancer patients [2].

There is a growing consensus that PGRMC1 is critical for the transport of specific receptors to the plasma membrane. The receptors include EGFR, GLP1R, glucagon-like peptide 1 receptor [16], and mPR1*α*, membrane progesterone receptor *α* [17]. PGRMC1 binds to mPR1*α* and transports it to the plasma membrane [17]. Indeed, PGRMC1 was originally identified as a putative hormone receptor or “receptor membrane component” [18]-[20]. Partially purified PGRMC1 binds to progesterone [21] [22], and recently, progesterone binding by recombinant PGRMC1 was reported [23], suggesting a direct role for PGRMC1 in progesterone function. PGRMC1 has an established role in progesterone signaling [5] [6] [24], and in some diseases, such as breast cancer, this contributes to hormonal growth and anti-apoptotic signaling [25]. However, PGRMC1 shares no homology with hormone receptors [26] but has motifs that are structurally related to cytochrome *b_5_*, and PGRMC1 binds heme [26]-[33], an evolutionarily conserved function [28] [32] [34] that is distinct from progesterone binding [23].

According to the cancer stem cell theory, tumors contain a sub-population of cells with extended replicative potential that contribute to drug resistance [35] [36]. Cancer stem cells are thought to arise from mutations to either normal stem cells or transit amplifying cells, with key signaling contributions from the tumor microenvironment. PGRMC1 is detectable in amniotic-derived mesenchymal cells [37] and has been identified as an important hormonal signaling intermediate in neuronal stem cells [24], but its expression and function in cancer-derived stem cells have not been determined.

We demonstrate here that PGRMC1 is elevated in multiple tumor types, including head and neck cancer and in oral cancer. Using immunohistochemistry of paraffin-embedded tissue, we also confirm previous findings from western blots of frozen tissue that PGRMC1 staining correlated with survival in lung cancer patients. According to the stem cell theory, cancer stem cells are critical for the long-term survival of a tumor population and its therapeutic resistance. We report here that PGRMC1 is abundant in lung cancer-derived stem cells from patients, and PGRMC1 inhibition triggered cell death in lung cancer stem cells where other therapeutic classes failed.

## 2. Methods

### 2.1. Tissue Arrays

The tissue arrays used were as follows. For head and neck cancers: HN483, HN242a and HN802 (US Biomax, Inc. Rockville, MD). For oral cancer, OR802 (US Biomax, Inc.). For lung cancer, BC04011, BC041114, BS04011, LC991 (US Biomax), IMH-305 and IMH-340 (Imgenex, Inc., San Diego, CA). For ovarian cancer, BC11115 and OV951 (US Biomax).

### 2.2. Immunological Reagents and Techniques

Tissue arrays were stained with an anti-PGRMC1 antibody raised to a recombinant protein composed of amino acids 43 - 195 of PGRMC1 (ProteinTech Group, Inc., Chicago IL). Immunohistochemistry was performed by the University of Kentucky Histology Laboratory using the Dako Envision kit (Carpinteria, CA) and following the manufacturer's instructions. Staining was analyzed and scored by a pathologist (Dr. Stewart), as well as other authors, and analyzed statistically using Microsoft Excel. Kaplan-Meier curves were analyzed and prepared using Graphpad Prism software. Blocking was performed with an equimolar concentration of purified, recombinant PGRMC1-glutathione S-transferase fusion protein spanning the antigenic region, and the protein has been described [27].

Protein levels were analyzed by western blot as previously described [27], blotting electrophoresed proteins to Immobilon-P membranes and developing using the West Pico chemiluminescent substrate (Pierce). Blots were performed at least in duplicate. The antibodies used were the following: anti-GAPDH (Santa Cruz, FL-335), anti-LCB (Cell Signaling, D11), anti-SQSTMI/p62 (Cell Signaling, 5114), anti-caspase 3 (Santa Cruz, sc-7138), anti-PARP (Santa Cruz, sc-7150) and anti-PGRMC1 [4].

### 2.3. Lung Cancer Stem Cells

Single-cell suspensions were isolated from patients at the Edwards Cancer Center using a gentleMACS Dissociator (Miltenyi, Auburn, CA), and C Tubes using a standardized, semi-automated protocol based on a combination of mechanical tissue disruption and incubation with a 50% solution 0.025% trypsin and Accutase (Innovative Cell Technologies, San Diego, CA). Cells were serially plated in 12-well, 6-well, 10-cm treated dishes and cultured to subconfluence in RPMI-1640 medium supplemented with 5% irradiated, heat inactivated, defined fetal bovine serum (Thermofisher/Hyclone), and 50 U of penicillin and 5 mg of streptomycin/mL of medium (Thermofisher/Mediatech). Cancer stem cells were selected and proliferated using a hydrodynamic focusing bioreactor as previously described [38]. After seven days of growth in the bioreactor, cells were then removed and counted using a CelloMeter automated counter and trypan blue exclusion to determine cellular viability and cell number. Cells were then immunophenotyped using fluoresceinated antibodies to CD133, CD44, CD24 and CXCR4 with an Accuri C-6 flow cytometer. For live cell assays, lung tumor-derived stem cells (positive for CD133, CD43, SSEA3/4, Oct4, alkaline phosphatase, aldehyde dehydrogenase and telomerase) were purchased from Celprogen, Inc. and were maintained in Human Lung Cancer Stem Cell Complete Growth Media with serum (Celprogen, Inc., Torrance, CA).

Cell viability was determined by Cell Titer Blue Cell Viability Assay (Promega). Erlotinib was from LC Laboratories (Woburn, MA), and other treatments were LY294002 (Sigma), PD98059 (Sigma) and AG-205 (Tocris). For drug treatments, cells were maintained in complete media and switched to serum free media (DMEM containing antibiotics) and various drugs for 24 hours. For microscopy, cells were treated with vehicle or 10 μM AG-205 for an additional 24 hours. Bright-field images were captured on a Nikon Eclipse TE200 microscope at 20× magnification. For viability assays, cells were treated for 72 hours in complete media, and viability was assayed according to the manufacturer's instructions. Absorbance was measured using a Spectra Max M2 spectrophotometer (Molecular Devices). For western blot analysis, cells were lysed in radioimmunoprecipitation buffer containing protease inhibitors and phosphatase inhibitors.

For dye exclusion, cells were trypsinized and treated with 5 μg/ml Hoechst 33342 (Sigma) at 37°C for 45 minutes. Cells were then washed with PBS (Gibco) and incubated in fresh media for 45 minutes at 37°C to allow for dye efflux before being centrifuged and analyzed again by FACS.

## 3. Results

### 3.1. PGRMC1 in Airway Cancers

**Figure 1:**
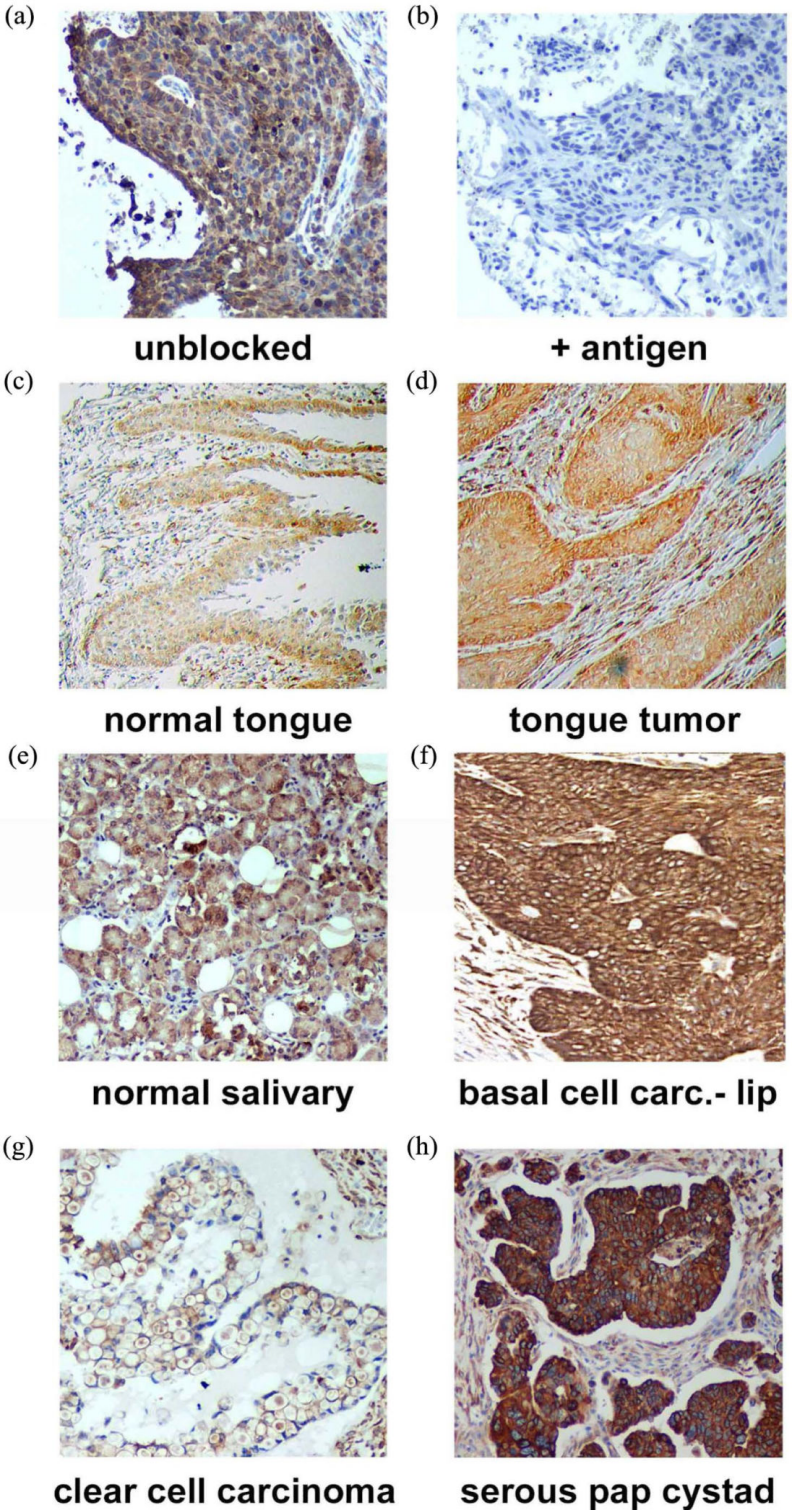
Immunohistochemistry of (a) a lung tumor section using the anti-PGRMC1 antibody 12990 from Proteintech Group, Inc.; (b) The same section stained with the same antibody plus recombinant PGRMC1 fusion protein; (c)-(h) PGRMC1 staining in multiple tissues, as indicated in the figure.

PGRMC1 is essential for lung tumor formation and metastasis, so we determined PGRMC1 levels by immunohistochemistry in 330 lung cancer samples using tissue microarrays, which included 58 patients with survival data. Tissues were stained for PGRMC1 and scored by a Pathologist. The antibody—generated to amino acids 43 - 195 of PGRMC1—was a commercial product from Proteintech Group, and to our knowledge, no controls for staining have been performed with this product. In staining of a tissue from an earlier study, we found that recombinant PGRMC1 fusion protein blocked staining with the antibody, suggesting that it is specific (**Figure 1(a)** and **Figure 1(b)**).

**Figure 2:**
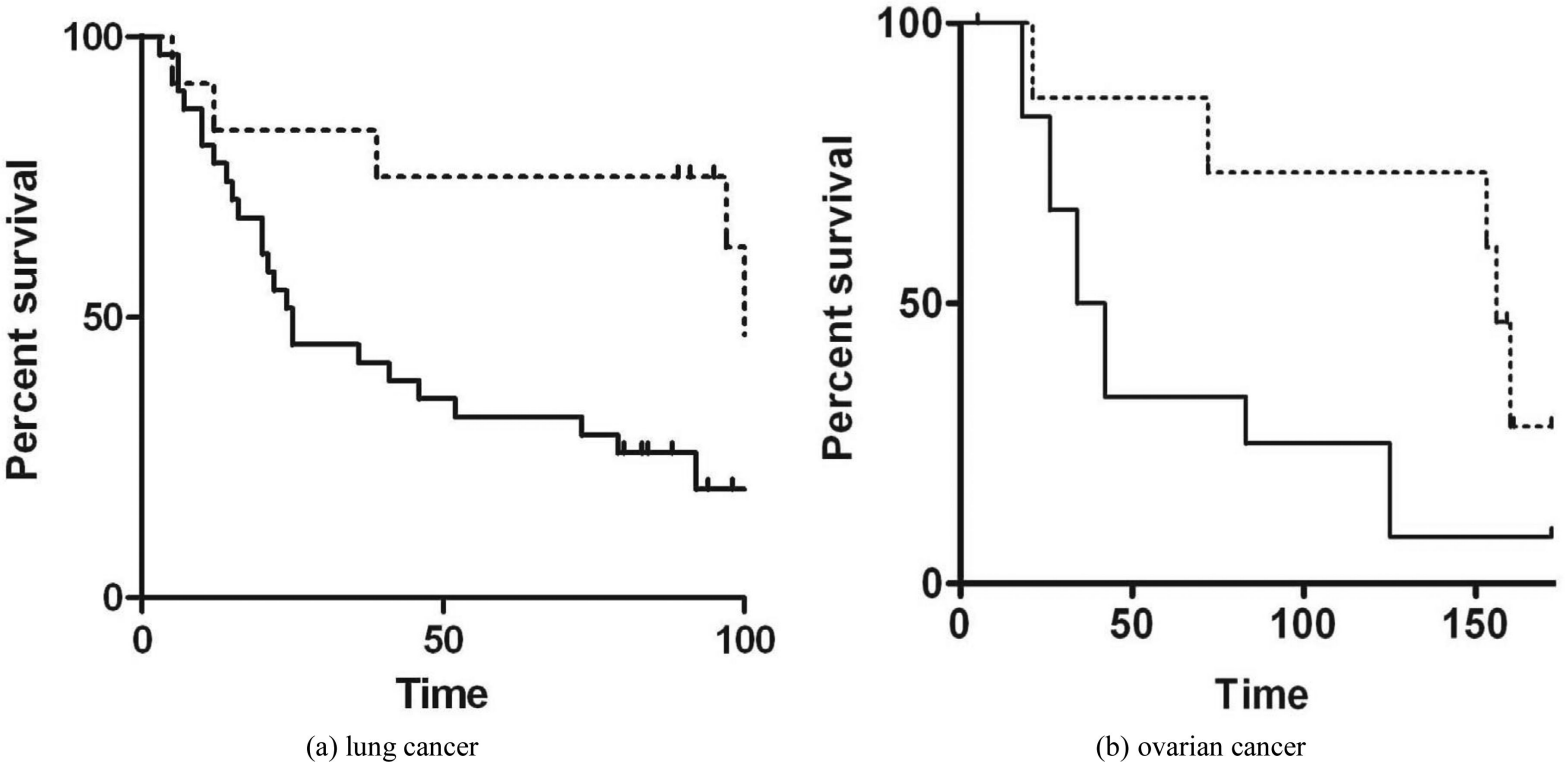
(a) Kaplan-Meier plot in samples from patients with lung cancer, comparing high PGRMC1 staining tumors (solid line) with low PGRMC1 staining tumors (dashed line); (b) a similar analysis to panel a, for ovarian cancers.

Consistent with earlier western blotting studies [2], PGRMC1 was significantly higher in lung tumors than normal tissue (p = 2 × 10^−7^, 2-sided t-test) and was higher in stage I tumors compared to stage II (*p* = 3 × 10^−5^, 2-sided t-test) but was not associated with age at diagnosis or gender. High PGRMC1-expressing tumors had a significantly worse overall survival (*p* = 0.02 by Gehan-Breslow-Wilcoxon test), with median survival times of 25 months for high expressers versus 108 for low expressers (**Figure 2(a)**).

PGRMC1 staining was analyzed in 173 tissues from the head and neck, including the oral cavity, in tissue microarrays. Tumors from multiple sites within the head and neck region stained strongly for PGRMC1 (**Figures 1(c)-(f)**), including tumors of the submaxilla, cheek, parotid gland, gingiva and larynx (**Supplemental Figure 1**). PGRMC1 was significantly elevated in all head and neck tumors (n = 173) compared to corresponding normal tissues (n = 23, *p* = 0.004, 2-sided *t*-test). There was no association with stage or gender, but PGRMC1 staining was higher in tumors from patients younger than 60 (*p* = 0.03, n = 113). For example, the highest levels of staining were located in the tongue, and PGRMC1 was elevated in tongue tumors (n = 16, **Figure 1(c)** and **Figure 1(d)**) relative to normal tongue tissue (n = 18, *p* = 0.009).

### 3.2. PGRMC1 in Ovarian Cancer

In ovarian tissues, PGRMC1 staining was not significantly different in normal ovarian tissue compared to tumor adjacent tissue, clear cell carcinomas or mucinous papillary adenocarcinomas (**Figure 1(g)**). In contrast, PGRMC1 was significantly elevated in serous papillary adenocarcinoma (*p* = 4 × 10^−5^), papillary serous cystadenocarcinoma (**Figure 1(h)**, *p* = 0.006) and endometroid carcinoma (*p* = 0.02, **Supplemental Figure 2**). PGRMC1 was also elevated in metastatic serous papillary adenocarcinoma compared to normal tissue (*p* = 0.003), but was not significantly different from the primary serous papillary adenocarcinoma. This difference was highly significant (**Figure 2(b)**, *p* = 0.0011 by Mantel-Cox test and 0.0014 by Gehan-Breslow-Wilcoxon test).

### 3.3. PGRMC1 in Cancer-Derived Stem Cells

**Figure 3:**
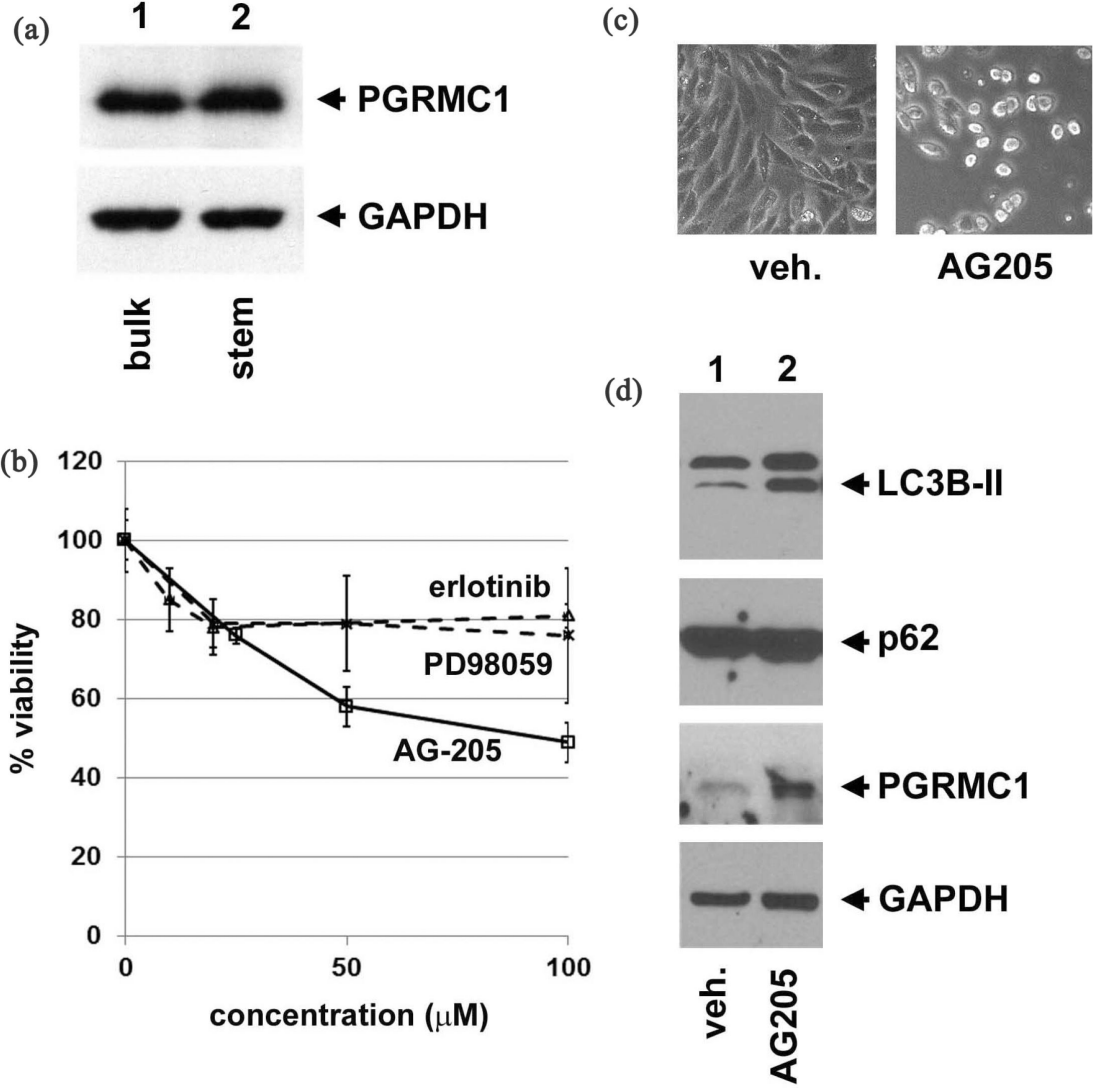
(a) Bulk tumor cells and tumor stem cells were isolated from a patient with lung cancer and analyzed by western blot for PGRMC1 (upper panel) and GAPDH (lower panel, loading control). PGRMC1 was abundant in both the bulk tumor cells and the stem cells; (b) Cancer-derived stem cells were treated with various ligands and assayed for viability. Neither the EGFR inhibitor erlotinib nor the ERK inhibitor PD98059 were active against the cells, while the PGRMC1 ligand AG-205 inhibited viability of the cells; (c) Bright field imaging of cancer-derived stem cells treated with AG-205 revealed cell rounding in the majority of the cells. d Western blot analysis of stem cells treated with vehicle (lane 1) or AG-205 (lane 2) revealed increased levels of the autophagy initiating protein LC3B-II (top panel, lower band), while the levels of the autophagy substrate p62 were unchanged (second panel). PGRMC1 levels increased following treatment (third panel), and GAPDH served as a loading control.

The association between PGRMC1 levels and tumors suggested a potential role for PGRMC1 in stem cell maintenance. Stem cells were isolated from surgical specimens at Marshall University using a PBRX bioreactor, and western blots revealed abundant PGRMC1 in isolated stem cells as well as in the bulk of the tumor (**Figure 3(a)**). The isolated stem cells were pre-screened for expression of CD133, CD44, CD24 and CXCR4 as described [38].

To assess the role of PGRMC1 in stem cell viability, we treated lung tumor-derived stem cells with the PGRMC1 ligand AG-205 [4], which had an IC_50_ of 95 μM (**Figure 3(b)**, solid lines), and induced cell rounding (**Figure 3(c)**). In contrast, stem cells were highly resistant to the EGFR inhibitor erlotinib, a widely used drug for lung cancer, and the ERK inhibitor PD98059, which is active against a variety of cancer cell lines (**Figure3(b)**, dashed lines). Because the cells rounded and detached, apoptosis is a potential cell death mechanism, but AG-205 treatment did not cause changes in markers of apoptosis, including cleavage of PARP and caspase-3 (**Supplemental Figure 3**). The ability to efflux dyes is a key marker of cancer stem cells, and we found that lung cancer stem cells had the properties typical of a “side scatter” population (**Supplemental Figure 4**). However, drug efflux did not change with AG-205 treatment.

In some cases, autophagy can be a cell death mechanism that acts as an alternative to apoptosis [39], and PGRMC1 has been implicated in autophagy [40], a metabolic process in which aged proteins and organelles are degraded in the lysosome [41]. LC3B is both a key mediator of autophagy and an autophagy substrate that is cleaved and lipidated to the mature LC3B-II form. We detected increased levels of LC3B-II upon PGRMC1 ligand treatment, suggesting that autophagy was induced (**Figure 3(d)**, top panel). PGRMC1 ligands induce autophagy in lung cancer but also arrest the process, so that autophagy substrates are not degraded. In stem cells, PGRMC1 ligand treatment increased LC3B-II levels but did not decrease levels of the autophagy substrate p62. The autophagy inhibitor chloroquine behaved similarly (data not shown), consistent with an autophagy arrest in cancer stem cells treated with PGRMC1 ligands. However, it is unlikely that autophagy arrest caused cell death, because chloroquine had no effect on cell viability. Notably, AG-205 caused an increase in PGRMC1 levels (**Figure 3(d)**, third panel), suggesting that AG-205 alters PGRMC1 stability.

## 4. Discussion

PGRMC1 is a membrane-associated protein implicated in the transport of multiple receptors to the plasma membrane, and multiple studies have proposed PGRMC1 ligands as therapeutics or diagnostic agents for cancer. In the present study, we demonstrate that PGRMC1 was elevated in a large cohort of lung tumors, which included multiple histological subtypes, and where it was associated with poor survival. To our knowledge, this is also the first report of PGRMC1 expression in the oral cavity, and we detected strong PGRMC1 expression in multiple organs, particularly in the tongue. In some tissues, PGRMC1 was enhanced on the outer surface of the tissue (**Figure 1(c)**), suggesting a role in secretion or trafficking. Indeed, PGRMC1 is secreted in lung tumors [2].

This is, to our knowledge, the first report of PGRMC1 expression in a relatively large cohort of ovarian cancers. PGRMC1 has been previously shown to play a key role in ovarian tumor growth, apoptosis resistance, invasion, angiogenesis, drug resistance and metastasis [5] [21] [42]-[45]. There are also numerous reports linking PGRMC1 to normal ovarian function, including follicle development [46] [47], and is aberrant in premature ovarian failure [48]. Thus, the finding that high PGRMC1-expressing tumors correlate with poor overall survival emphasizes the importance of PGRMC1 as a therapeutic target in ovarian cancer.

The current study is, to our knowledge, the first report of PGRMC1 expression in cancer stem cells. PGRMC1 was detected in stem cells isolated using two different approaches, and the second commercial source was verified by measuring dye exclusion. The ability of the PGRMC1 ligand AG-205 to induce rounding and inhibit viability in the stem cells differed markedly from known inhibitors. The mechanism through which PGRMC1 functions in the cells is enigmatic, because EGFR inhibition had no effect on tumor stem cell growth, and the cells did not die from apoptosis. We were also unable to detect changes in signaling (*i.e.* global tyrosine phosphorylation), protein degradation (ubiquinated protein levels) and PGRMC1-sensitive metabolism (INSIG-1 and CYP51 levels). AG-205 treatment induced the early event of autophagy, LC3-II accumulation, but autophagy inhibition with chloroquine did not affect viability, suggesting that other mechanisms are at work. We are currently investigating candidate mechanisms in signaling and metabolism.

PGRMC1 was identified as the sigma-2 receptor in 2011, and there is an ongoing, vigorous debate as to whether the proteins are identical. The sigma-2 receptor (S2R) is a membrane-associated protein that binds a number of pharmacological compounds, including signature ligands SV-119, siramesine, SM-21 and PB-28 [49]-[52], among others, as well as multiple anti-depressants and stimulants. S2R and PGRMC1 have very similar patterns of being induced in cancer, and S2R is detectable in stem cells. Xu, *et al*. identified PGRMC1 based on its ability to bind and cross-link to the ligand WC-21 [53]. The sigma-2 receptor probe RMH-4 was competed by classical S2R ligands, such as DTG, siramesine, SV119 and WC-26, and equally by the PGRMC1 ligand AG-205 [53]. Furthermore, RNAi knockdown of PGRMC1 in HeLa cells decreased binding of labeled RMH-4, while PGRMC1 over-expression increased binding [53].

In contrast to the reports of the Mach group and co-workers, Abate, *et al*. demonstrated that PGRMC1 knockdown in MCF-7 breast cancer cells did not affect DTG (1,3-di-o-tolylguanidine) binding in membrane fractions [54]. At the moment, it is difficult to reconcile these findings, except that they were performed with different cell lines and ligands. In addition, Chu, *et al*. recently showed that PGRMC1 knockout in NSC34 cells did not affect binding of DTG or photolabeling of the 18 kDa “S2R” with [^125^I]-Iodo-azido-fenpropriomorph. Based on these findings, the authors stated in the title of the paper that “The sigma-2 receptor and the progesterone receptor membrane component 1 are different binding sites derived from different genes”. There are some caveats. NSC34 cells are a hybrid of mouse embryonic spinal cord cells and mouse neuroblastoma cells and represent a very specialized example of cell biology, as do HeLa and PC12 cells.

As a compromise, it could be argued that the sigma-2 receptor is a separate entity from PGRMC1, but S2R-ligand binding requires PGRMC1, perhaps due to a direct interaction between S2R and PGRMC1. However, this is not consistent with the ability of IAF to photolabel S2R equally in control and PGRMC1-knockout cells. Another possibility is that the original probe, WC-21, has non-S2R binding activity, including binding to a PGRMC1 complex. However, the ability of multiple well characterized S2R ligands to efficiently compete for WC-21 binding argues against this possibility [53]. Clearly, there are many questions to be addressed in this field, including the identity of the 18 kDa “S2R” protein, and we have used the nomenclature “PGRMC1” in this study until the situation is resolved.

In summary, the present findings implicate elevated PGRMC1 expression in a broad range of tumor types. Tumors of the upper airways are intriguing for therapeutics, because topical delivery systems can allow greater penetrance of chemicals and biologicals targeting PGRMC1. In addition, we provide evidence linking PGRMC1 expression to cancer stem cells, which are notorious for their resistance to therapeutics, and the cancer stem cells in this study were highly resistant to powerful agents such as erlotinib and PD98059. In contrast, PGRMC1 inhibitors had activity against cancer stem cells, suggesting a role for PGRMC1 in maintaining cancer stem cell viability. In contrast, PGRMC1 inhibitors had activity against cancer stem cells, suggesting a role for PGRMC1 in cancer stem cell maintenance and the importance for inhibitors such as AG-205 for future therapeutics targeting cancer stem cells.

## 5. Conclusion

PGRMC1 is broadly expressed in a variety of tumors, where its expression is elevated in comparison to corresponding normal tissues. In some diseases, PGRMC1 expression correlates with poor patient survival, while in breast cancer, the correlation between PGRMC1 and survival is more complex and may depend on the patient population or epitopes being analyzed. PGRMC1 was expressed in two different patient-derived tumor stem cell populations and was required for viability in those cells. The results support PGRMC1 as a tumor biomarker and therapeutic target for multiple types of cancer.

## Supplementary Material

01
