# Supplementary material for: PGRMC1 Elevation in Multiple Cancers and Essential Role in Stem Cell Survival
Source: Adv Lung Cancer (Irvine). Author manuscript; Available in PMC 2016 Nov 17. (PMC5113835; doi:10.4236/alc.2015.43006)
Supplement: 01 [file NIHMS825997-supplement-01.pdf]

Supplemental Figure

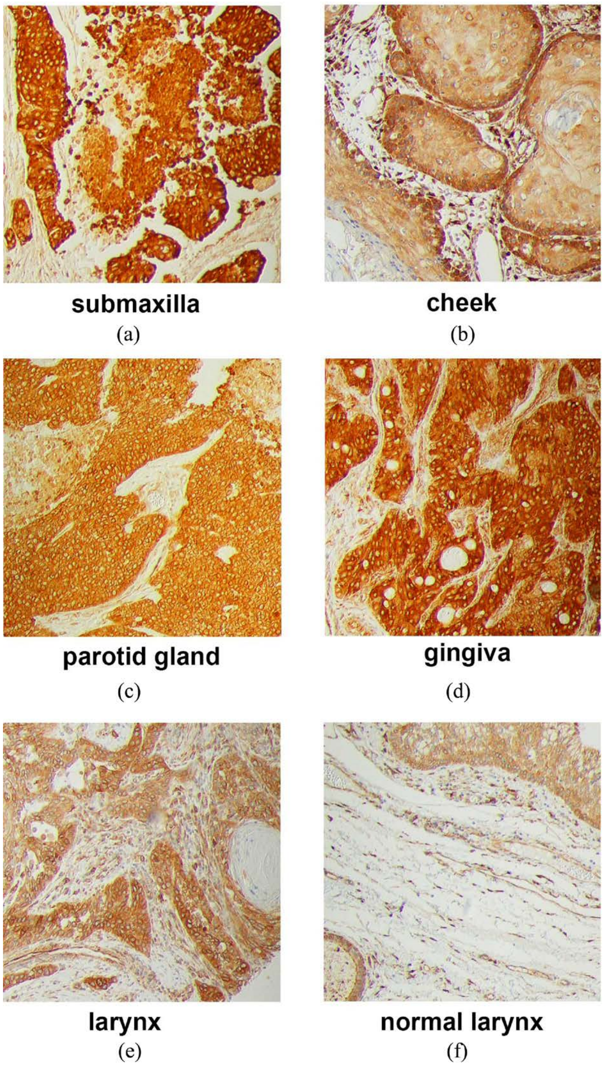

Supplemental Figure 1. Immunohistochemistry of tumors and tissue from the head and neck region (a) squamous cell carcinoma of right submaxilla, stage II; (b) squamous cell carcinoma of left cheek, stage II; (c) squamous cell carcinoma with necrosis of right parotid gland, stage II; (d) mucoepidermoid carcinoma of gingiva, stage I; (e) squamous cell carcinoma of larynx, stage IV; (f) normal larynx tissue.

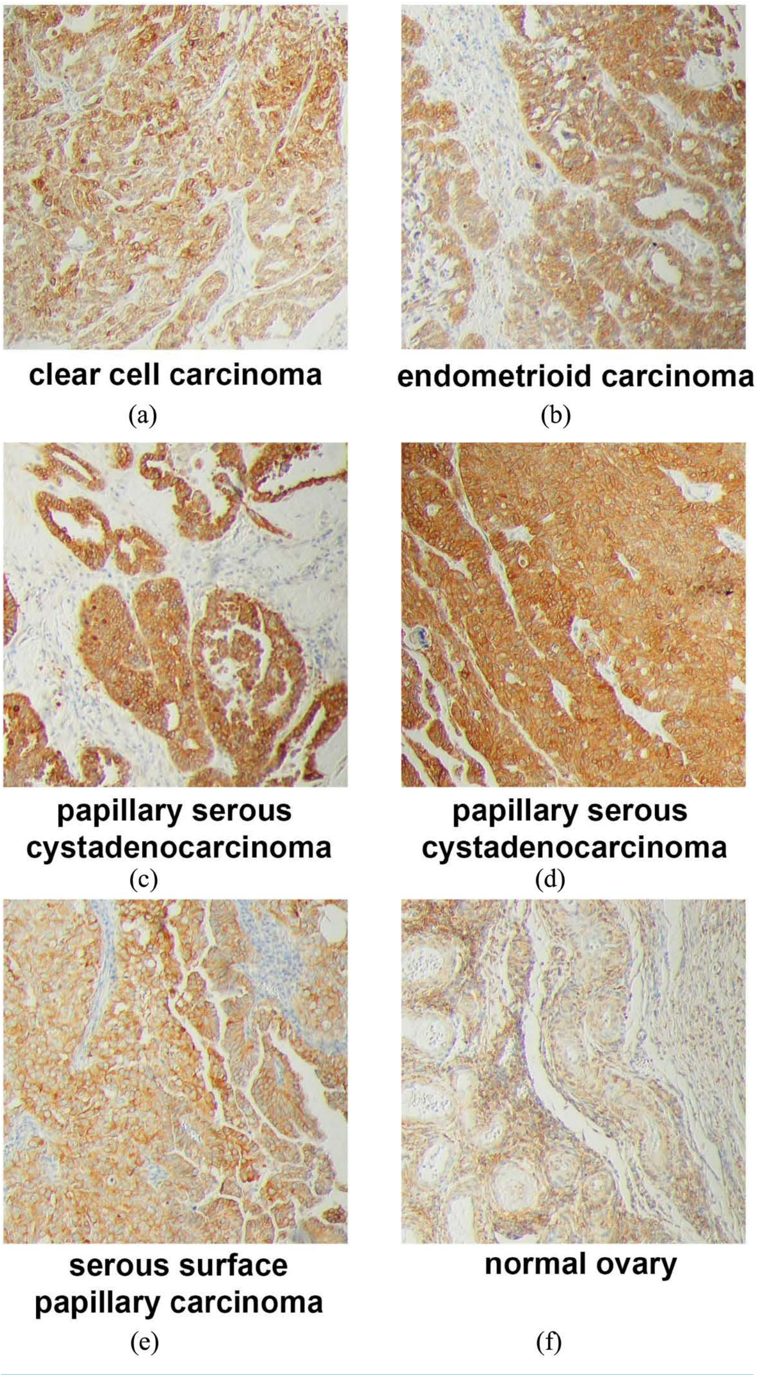

Supplemental Figure 2. Immunohistochemistry of ovarian tumors and tissue. (a) Clear cell carcinoma, stage IIB; (b) endometrioid carcinoma, stage IIB; (c) papillary serous cystadenocarcinoma, stage IIB; (d) papillary serous cystadenocarcinoma, stage IIIC; (e) serous surface papillary carcinoma; (f) normal ovary tissue.

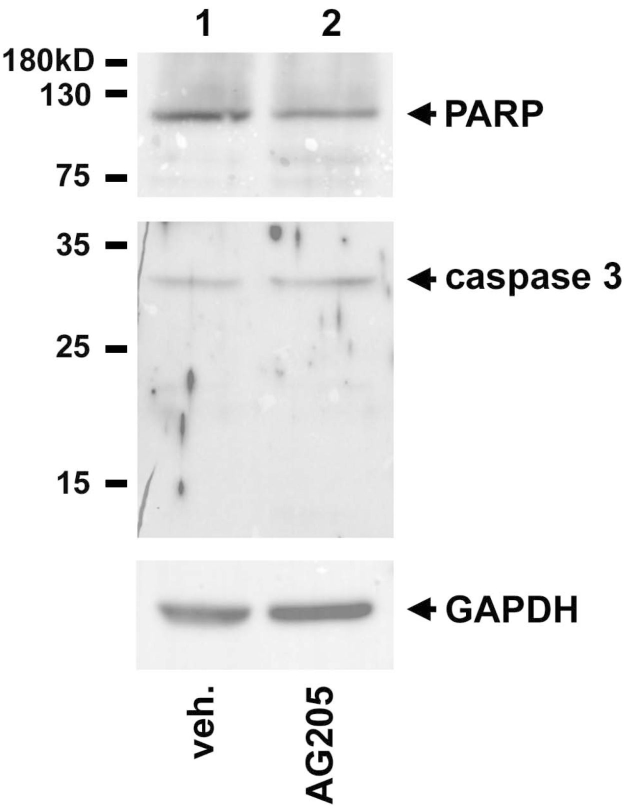

Supplemental Figure 3. Western blot analysis of stem cells treated with vehicle (lane 1) or 10 μM AG-205 (lane 2), as described in the methods section. Treatment with PGRMC1 ligand AG-205 did not induce cleavage of PARP (top panel) or caspase-3 (middle panel), which are common apoptosis markers. GAPDH (bottom panel) served as a loading control.

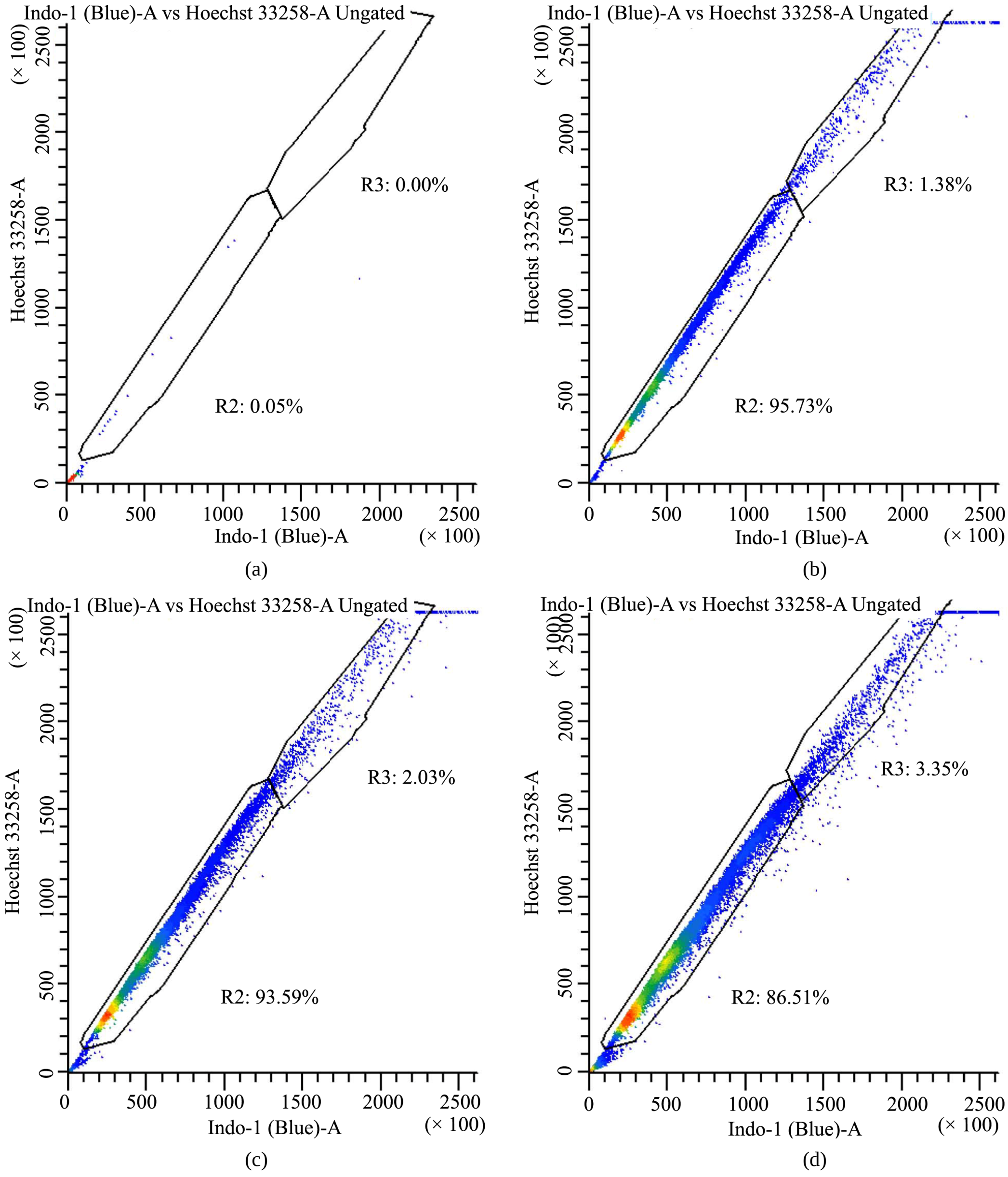

Supplemental Figure 4. Drug efflux pattern of lung cancer stem cells follows typical "side scatter" population. Lung cancer-derived stem cells were treated with vehicle control (panel a) or Hoechst 33342 (panels (b)-(d)). In panel (b) there were no further treatments, whereas cells were treated with 50 μM AG-205 for 24 hours in panel (c) or 48 hours in panel (d) in triplicate measurements, drug treatment did not affect dye exclusion to a significant extent.
